# Expectant Medicine

**Published:** 1865-12

**Authors:** 


### Editorial Department.

### Expectant Medicine.

In our last issue we noticed that the Massachusetts Medical Society have offered a prize for the best dissertation upon "*Expectant Medicine—the extent to which it is practiced at the present day, and the modes in which it is disguised or counterfeited.*" We would call attention not only to this opportunity of obtaining a prize, but to the other advantages it affords; and it might be well if the attention of some outsiders who do not read medical journals very much, could be attracted, for we believe them, more likely than any attentive readers, to be benefited by attention to this subject; it is eminently worthy the attention of practitioners generally, whereby some benefits might accrue from seriously thinking upon it, even though conclusions were unwritten and prize unobtained. We have considered our own chances for a prize, and were it not for the fact that "prize essays" are to be furnished monthly, for this Journal, to obtain which the "expectant" plan has never proved successful, we should most certainly forward our manuscript, of course "keeping dark" until after the prize was awarded.

*Expectant medicine and our means of disguising it*, is a free translation we prefer to make of the original text, not being however, positively informed that this is literal rendering of the prize proposition. Expectant medicine is understood to mean, giving nothing, administering it in the form of a pill or powder—pacifying the patient while nature cures the disease. That such a subterfuge has ever been practiced by the members of an honorable profession, we are sorry to be obliged to confess, but we have no "modes of disguising it." The impostor who pretends to cure disease with inert or absurd compounds we deridingly call a quack, and despise him as we do a thief or pickpocket. If physicians pretend by words or actions, to cure disease which they know terminates spontaneously in recovery, and administer inert and useless remedies to cover a truth they foolishly desire to conceal, in what do they differ from the mountebank and charlatan? Do physicians hesitate to tell their patients the truth concerning their maladies and the value of remedies in removing them? Are they not ready to go to them, with the glad truth, that nature favors their recovery, rather than whine out a doleful dependence upon drugs? Sometimes it might appear best for patients not to know the whole truth as to the nature of their diseases; but if we withhold opinions we deem better not to express, is it any reason for expressing others which we do not entertain?

Late in the history of medicine it was discovered that many diseases disappear without the aid of drugs, and that but comparatively few which have not this natural tendency to recovery can be thus terminated by medicine. This truth has been covered to some extent from a fear that medicine would suffer in its reputation for good; and thus one of the most grand discoveries ever made, and, withal, one which affords the strongest confidence to the educated physician, has been kept back from the people, and when they have "asked bread, we have given them a stone." This tendency of disease to terminate in recovery is one of the central truths of the healing art, around which a great many lesser truths cluster, and upon which they depend. That medicine is almost always capable of affording some relief, and is indispensable to the comfort and care of the sick is one of the lesser truths, but has been magnified so as to almost overshadow everything greater, better, and more exactly true. For a physician to prescribe medicine for the really sick, is a very important undertaking; the public may eat it with the relish of an epicure; ignorant pretenders who know nothing of the nature of disease or the therapeutical effects of drugs, can recommend them with impunity, but the physician acts under a most solemn obligation. People who are not much unwell may take considerable medicine, with comparative impunity, but those who are sick, whose organs of elimination are deranged or already overtaxed suffer more seriously, therefore it should be the crowning ambition of a physician to know how little is required, rather than how much can be borne. Why attempt to keep up a show of medication after the substance is no longer required? Are the people unwilling to know that many diseases terminate by natural recovery, uninfluenced by medicine? We were about to answer no, a thousand times no, but we must confess that eighteen hundred years of false reasoning, false teaching and false practice, has made many people rather unwilling to recover from disease without the assistance of drugs. This recovery without medicine is a new idea to mankind generally, and we are sorry to add, to a great many in the profession, and it requires time and honest dealing to change the current of popular thought and convince mankind of errors which have been incorporated into the constitution by centuries of "disguised and counterfeited medication."

We hope that no one will infer that we would underrate the value of medicine, while we deprecate this sort of practice, which disguises itself or counterfeits that which is genuine. We believe that we have a few medicines which are of great value in the treatment of disease, that our art of diagnosis has already reached a perfection which may well make us proud of our profession, and that there is no reason for making any false pretenses, disguising our practice, or counterfeiting a practice which we do not fully adopt.

It is probable that our Massachusetts friends designed to obtain something of a review of the various systems of imposition, which pass as systems of medical practice—systems which counterfeit and disguise as a trade, but for the present we have preferred to consider only the practice of the profession, and let the pretenders look out for themselves until we come to deal with them as they deserve.

